# Synthesis of Chitosan Beads Incorporating Graphene Oxide/Titanium Dioxide Nanoparticles for In Vivo Studies

**DOI:** 10.3390/molecules25102308

**Published:** 2020-05-14

**Authors:** Carlos David Grande Tovar, Jorge Iván Castro, Carlos Humberto Valencia, Paula A. Zapata, Moisés A. Solano, Edwin Florez López, Manuel N. Chaur, Mayra Eliana Valencia Zapata, José Herminsul Mina Hernandez

**Affiliations:** 1 Programa de Química, Universidad del Atlántico, Carrera 30 Número 8-49, 081008 Puerto Colombia, Colombia; madolfosolano@mail.uniatlantico.edu.co; 2 Grupo de Investigación SIMERQO, Departamento de Química, Universidad del Valle, Calle 13 No. 100-00, 76001 Cali, Colombia; jorge.castro@correounivalle.edu.co; 3 Grupo Biomateriales Dentales, Escuela de Odontología, Universidad del Valle, Calle 4B # 36-00, 76001 Cali, Colombia; carlos.humberto.valencia@correounivalle.edu.co; 4 Grupo de Polímeros, Facultad de Química y Biología, Universidad de Santiago de Chile, USACH, Casilla 40, Correo 33, 9170020 Santiago, Chile; paula.zapata@usach.cl; 5 Grupo de Investigación en Química y Biotecnología QUIBIO, Universidad Santiago de Cali, Calle 5 No 62-00, 760035 Cali, Colombia; edwin.florez00@usc.edu.co; 6 Centro de Excelencia en Nuevos Materiales (CENM), Universidad del Valle, Calle 13 No. 100-00, 760032 Santiago de Cali, Colombia; 7 Escuela de Ingeniería de Materiales, Facultad de Ingeniería, Universidad del Valle, Calle 13 No. 100-00, 760032 Santiago de Cali, Colombia; valencia.mayra@correounivalle.edu.co (M.E.V.Z.); jose.mina@correounivalle.edu.co (J.H.M.H.)

**Keywords:** chitosan beads, graphene-oxide, titanium dioxide nanoparticles, nanocomposites, tissue engineering

## Abstract

Scaffold development for cell regeneration has increased in recent years due to the high demand for more efficient and biocompatible materials. Nanomaterials have become a critical alternative for mechanical, thermal, and antimicrobial property reinforcement in several biopolymers. In this work, four different chitosan (CS) bead formulations crosslinked with glutaraldehyde (GLA), including titanium dioxide nanoparticles (TiO_2_), and graphene oxide (GO) nanosheets, were prepared with potential biomedical applications in mind. The characterization of by FTIR spectroscopy, X-ray photoelectron spectroscopy (XRD), thermogravimetric analysis (TGA), energy-dispersive spectroscopy (EDS) and scanning electron microscopy (SEM), demonstrated an efficient preparation of nanocomposites, with nanoparticles well-dispersed in the polymer matrix. In vivo, subdermal implantation of the beads in Wistar rat′s tissue for 90 days showed a proper and complete healing process without any allergenic response to any of the formulations. Masson′s trichrome staining of the histological implanted tissues demonstrated the presence of a group of macrophage/histiocyte compatible cells, which indicates a high degree of biocompatibility of the beads. The materials were very stable under body conditions as the morphometry studies showed, but with low resorption percentages. These high stability beads could be used as biocompatible, resistant materials for long-term applications. The results presented in this study show the enormous potential of these chitosan nanocomposites in cell regeneration and biomedical applications.

## 1. Introduction

Due to many losses of organs in people during sickness or accident events, tissue engineering has gained significant ground as researchers seek alternatives for scaffolding design that promote cell adhesion, proliferation, and differentiation. Scaffold design (including porosity and interconnection) are of great importance due to their influence in cell migration, attachment, and proliferation [1]. A large surface area and high interconnected porosity must be available to allow nutrient transportation, waste disposal, cell infiltration, neovascularization, and cell proliferation [2,3,4].

The use of biopolymers is preferred over synthetic versions, although they have a lower cost and more straightforward synthetic modification to improve biocompatibility and cell adhesion [5]. Despite the enormous diversity in the artificial polymer market, natural counterparts offer many advantages for application in tissue engineering [6]. The benefits of using biopolymers are their high biocompatibility, hydrophilicity, and lower toxicity than their synthetic counterparts. However, biopolymers like polysaccharides have low mechanical properties due to their intrinsic hydrophilicity, which makes them highly biodegradable. 

Among biopolymers, chitosan (CS) is one of the most used polysaccharides, which has gained much attention for biomedical applications due to its biocompatibility and biodegradability. There are several applications of chitosan and nanochitosan preparations in tissue engineering, thanks to their excellent cell adhesion and low toxicity [7,8,9,10,11].

Chitosan was used for wound healing applications with reinforcement using different nanoparticles [12]. A comprehensive review has been published about future trends of this topic [13]. The use of chitosan-based nanoparticles in tissue engineering, cancer therapy, gene therapy, and drug-delivery has also been reviewed, with a particular focus on toxicity/safety of using animal tissues and the bactericidal properties of chitosan [14].

Nanotechnology offers solutions to the inconveniences of low durability of biopolymers [15], for example, in biomedicine [16]. Our group has obtained promising biocompatibility and long-term stability results with chitosan (CS)/polyvinyl alcohol (PVA) nanocomposites incorporating carbon nanomaterials (graphene oxide and carbon nano-onions) [17,18,19]. 

Titanium-related composites are preferred for biomedical applications due to their excellent biocompatibility, resistance to body fluids, mechanical properties, anti-corrosion capacity, and flexibility [20,21]. However, their properties depend on the surface area, which focuses considerable attention on the synthesis of titanium-derived nanomaterials [22]. Titanium oxide (TiO_2_) has been synthesized for a wide variety of applications [23]. For example, in bone replacement, where it provides biocompatibility and preventing bacterial adhesion [24,25]. 

Likewise, the use of graphene has also skyrocketed in recent years thanks to the new properties it exhibits like low thickness (one to ten layers), high flexibility, and strong-resistance supported by the σ type bonds in the carbon two-dimensional crystal lattice sp^2^ structure [26]. This planar, cyclic, and highly conjugated (through π electron resonance) structure, offers an improved alternative to immobilize fluorescent probes and cells for bioimaging/biosensing [27,28] and disease diagnostics [29,30].

Graphene also presents high stability due to its flat aromatic polycyclic structure [31]. However, graphene is hard to handle, and chemical modifications are needed to improve biocompatibility and dispersibility in aqueous media, a key factor for heavy metal adsorption in water treatment applications [32].

Chemical oxidation to produce graphene oxide, introduces hydroxyl (OH), epoxy (COC), and carboxyl (COOH) functions on the surface, reinforcing the ability to interact with other molecules chemically (such as chitosan), especially by hydrogen bonding. In parallel hydrophilicity is simultaneously introduced to the substantial hydrophobic nature of the sp^2^-carbon network [33,34]. Hydrophilic pH-dependent negative hydroxyl groups are essential as anchoring points for chemical functionalization, colloid-based and drug-delivery applications [35]. At the same time, the hydrophobic nature of the carbon backbone is essential for binding proteins and lipids through the hydrophobic domain of the molecules, which is useful in molecule-carrier applications or for antimicrobial properties [36].

In comparison to other nanomaterials, GO offers several advantages for biomedical applications such as low-cost and safer preparation with lesser accumulation of toxic metallic impurities. Also, the extensive surface area is accessible for molecular interactions, significant for a highly-efficient drug loading [37]. Besides, GO introduction to nanocarriers and scaffolds results in antimicrobial properties and improved tumor-passive targeting effect with a higher tumor uptake capacity, very interesting for anticancer therapy, thanks to its enhanced permeability and unique structure [38]. 

The antibacterial properties of GO are based on the bacterial cells’ membrane rupture caused by the sharp structure of GO and destructive extraction of lipid molecules, but also on the multiple-cell functions affected by oxidative stress of proteins, enzymes, and DNA that react with reactive oxygen species [39].

GO introduction to scaffolds and biomedical devices also provides thermal and mechanical reinforcement. For example, GO is introduced in bone-cements for mechanical support [40]. However, there is no consensus on whether GO introduction causes cytotoxicity. Some studies have stated that no cytotoxicity is produced [41,42,43]. In contrast, others have pointed out that a high level of cytotoxicity is introduced, especially when micro-sized GO and not nano-sized GO is added [37].

CS beads have been prepared to investigate their in vivo and in vitro biodegradation [44,45,46]. Lim et al. studied the influence of the degree of deacetylation and porosity on the in vivo and in vitro biodegradation of beads [47]. Kim et al. reported by in vitro studies the increase in cell proliferation and production of an extracellular matrix using a chitosan scaffold containing transforming growth factor-β1 (TGF-β1) [48].

Although some studies have focused on the development of chitosan composites/nanocomposites for biomedical applications [14,49,50], the low stability and the lack of in vivo study information is evident. On the other hand, ternary CS-GO-TiO_2_ systems which could combine the biocompatibility of the three components and the reinforcement effect under physiological conditions, to produce biocompatible and resistant components for long-term applications, have not been reported.

The requirement for improvement in the stability and biocompatibility under physiological conditions to prolong the durability of the nanocomposites with three-dimensional porous structures remains latent. In the literature, there is still a strong need to determine whether the GO functionalization or combination with other components improves the biocompatibility, especially with long-term in vivo and toxicity studies [37].

In the present work, beads of CS incorporating GO, TiO_2_ and both nanomaterials were synthesized and chemically and thermally characterized. At the same time, the biocompatibility was assayed by subdermal implantations in Wistar rat tissues for 90 days. Based on their morphology, biocompatibility, and long-lasting durability the present beads could be promising in long-term applications like bone-tissue engineering.

## 2. Results and Discussion

### 2.1. Characterization of Spherical Titanium Oxide Nanoparticles

**Figure 1:**
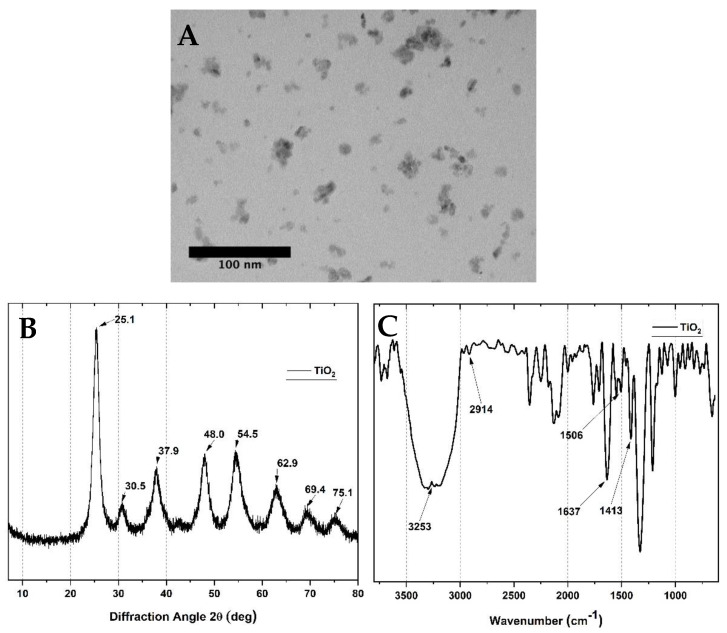
Characterization of TiO_2_ nanoparticles by (**A**) TEM, (**B**) XRD, and (**C**) FTIR spectroscopy.

Spherical TiO_2_ nanoparticles were prepared according to a previously reported methodology [51]. The morphology and size studies of the TiO_2_ nanoparticles by TEM (Figure 1A) revealed spherical characteristics, with a mean diameter of ~10 nm and low dispersion. The crystallinity of the TiO_2_ was analyzed by the XRD technique (Figure 1B). The diffractograms show 2θ values at 25.1, 37.9, 48.0, 54.5, 62.9, 69.4, and 75.1°, corresponding to (101), (004), (200), (211), (204), and (220) reflection planes of TiO_2_ [51]. On the other hand, FTIR analysis of the TiO_2_ particles (Figure 1C) presented a strong band corresponding to OH at 3253 cm^−1^. The 1637 cm^−1^ OH band is usually attributed to adsorbed water molecules. The bands of the CH_2_ groups appear at 2914 cm^−1^. The CH_2_ scissoring bands were also observed at 1413 cm^−1^.

### 2.2. GO Characterization 

**Figure 2:**
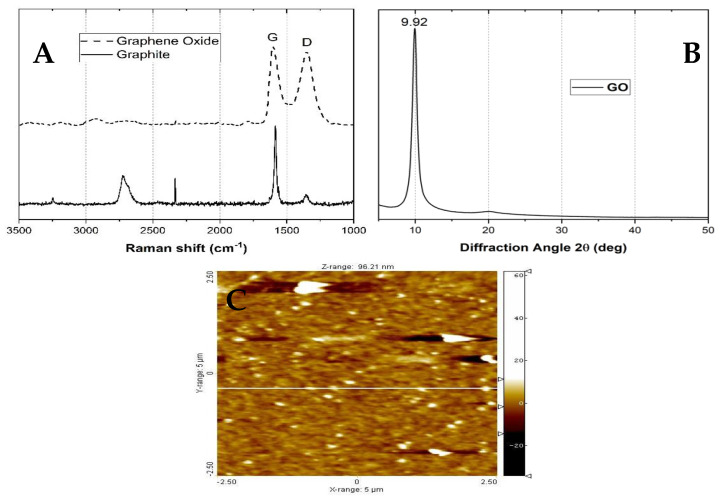
Characterization of GO by (**A**) Raman spectroscopy, (**B**) XRD, and (**C**) atomic force microscopy (AFM).

Raman spectroscopy (Figure 2A) shows two broad peaks between 1300 and 1600 cm^−1^, which are related to the graphitic structure. Usually, the degree of the disorder of the carbon structure is related to the sp^2^ and sp^3^ carbon atom ratio, obtained from the D and G band intensities. The band at 1580 cm^−1^ (the G band) corresponds to the E_2g_ mode of sp^2^ hybridized carbons. On the other side, the D band is related to the breathing mode of the sp^2^ carbon rings, which is activated by the introduction of carbon functionalization (oxidation) and a higher amount of sp^3^ hybridized carbons [52]. 

Moreover, X-ray diffraction patterns of the GO (Figure 2B) showed at 2θ = 9.92° a characteristic peak due to the scattering of the -COOH introduction in GO sheets [53]. The absence of peaks between 2θ = 20–50° indicates an excellent exfoliation process for graphite oxide [33]. When preparing the scaffolds, the exfoliation process allows the graphite oxide to be dispersed, separating the sheets, and reshaping the GO. Applying Bragg′s law to calculate the interlaminar distance for GO, and increasing from 3.36 Å to 9.05 Å was estimated after the oxidation process, similar to previous studies which will facilitate the dispersion in the aqueous media [54,55]. Atomic force microscopy (AFM) studies (Figure 2C) confirmed the low thickness obtained (from the calculation of the roughness, approximately 26.90 ± 2.12 nm), measurements obtained after GO exfoliation in solution. 

In general, a smooth morphology with low thickness was the average observation (smaller than 10 nm). However, some aggregates of 20–30 nm were observed due to the cumulation of some graphene layers [33]. 

In general, a smooth morphology with low thickness (smaller than 10 nm) was observed. However, some aggregates of 20–30 nm are apparent due to the cumulation of some graphene layers [33]. Still, the average size is meager, confirming an efficient exfoliation process of the GO sheets.

### 2.3. Bead Characterization

#### 2.3.1. Fourier Transform Infrared Spectroscopy (FTIR)

**Figure 3:**
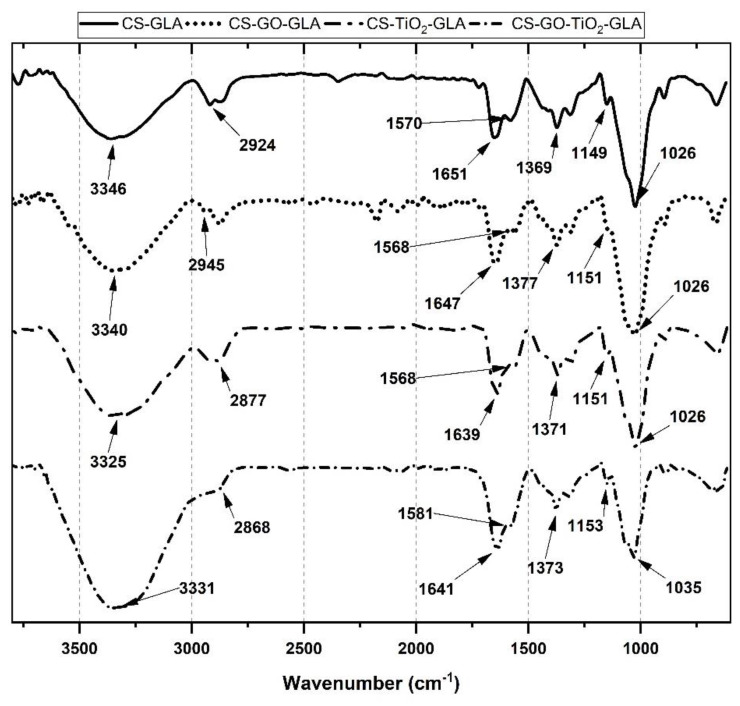
FTIR images of the different nanocomposite beads of CS-GLA, CS-GO-GLA, CS-TiO_2_-GLA, and CS-TiO_2_-GO-GLA).

Figure 3 shows the FTIR spectrum of the CS nanocomposite beads. CS-GLA contains all the characteristic bands of CS, such as that at 3346 cm^−1^, due to the O-H and NH_2_ overlapped bands [56]. Amino groups of chitosan reacted with glutaraldehyde (GLA), generating imine groups (C=N) which produce a corresponding imine band at 1651 cm^−1^. At 2924 cm^−1^, the -CH stretching vibration of -CH and -CH_2_ was observed, while the -CH symmetric bending vibrations of -CHOH- are also apparent at 1373 cm^−1^. At 1149 and 1026 cm^−1^, the -CO stretching vibration in -COH is present. The new band at 1570 cm^−1^ for CS-GLA beads is due to the new C=C bonds formed after the crosslinking reaction between CS-GLA [57]. On the other hand, the low intensity of the 1420 cm^−1^ band could indicate that primary amino groups are the main responsible for the crosslinking reaction with GLA [57,58]. It is also essential that there are no bands at 1720 cm^−1^ due to unreacted aldehyde groups. For the CS-GO-GLA, the broadening of the O-H band is due to the presence of the GO. However, there is no presence of bands due to the C=O stretching bond of the low amount of GO added. Finally, CS-TiO_2_-GLA and CS-TiO_2_-GO-GLA spectra show related groups with O-H band shifting to 3331 cm^−1^ and the broadening of the bands due to the O-H (3331 cm^−1^) and C-O-C bonds of the oxygenated groups (1026 and 1035 cm^−1^). 

#### 2.3.2. X-ray Diffraction (DRX)

**Figure 4:**
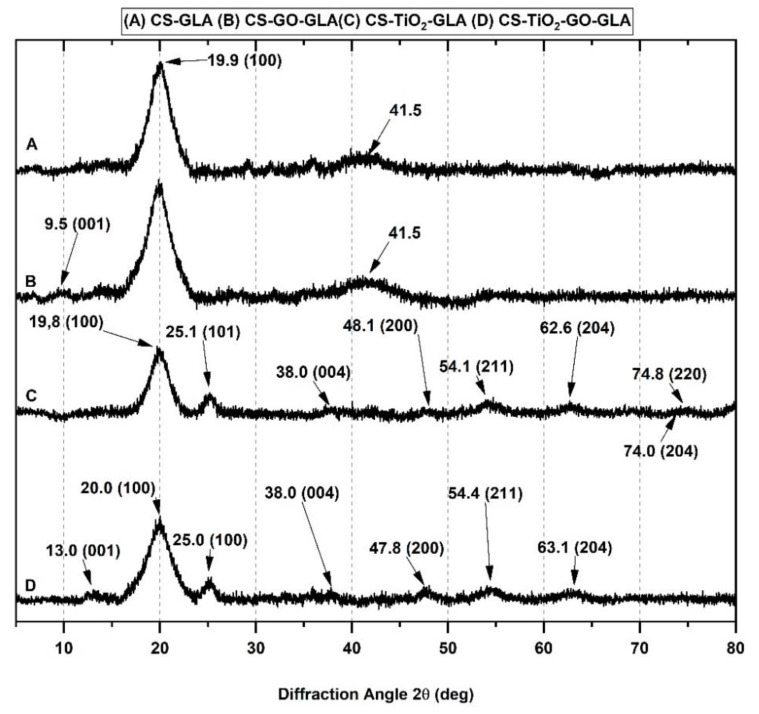
X-ray diffraction (DRX) of the different nanocomposite beads: (**A**) CS-GLA, (**B**) CS-GO-GLA, (**C**) CS-TiO_2_-GLA, and (**D**) CS-TiO_2_-GO-GLA.

XRD studied the crystallinity of the nanocomposite beads. Pure CS-GLA (Figure 4A) shows a peak at 2θ = 19.9°, which corresponds to the 100 strong reflections [59]. Six polymorphs have been proposed for chitosan: “tendon chitosan,” “annealed,” “1-2”, “L-2”, “form-I” and “form-II” according to previous studies [60,61,62]. The form I crystal is orthorhombic with a unit cell of a = 7.76, b = 10.91, and c = 10.30 Å. The form II crystal is also orthorhombic with a unit cell of a = 4.4, b = 10.0, and c = 10.3 Å (fiber axis). For CS-GO-GLA (Figure 4B), peak at 9.5° corresponds to a well-dispersed GO sheet and corresponds to the reflection of the (001) plane [63]. The other peak at 2θ = 41.5° corresponds to the CS crystalline structure. The CS-TiO_2_-GLA diffractogram (Figure 4C) shows peaks at about 25.1°, 38.0°, 48.1°, 54.1°, 62.6°, 74.0°, and 74.8°, corresponding to the (101), (004), (200), (211), (204), and (220) tetragonal crystal planes of the anatase phase of TiO_2_ [51,64,65,66,67]. The peak at 2θ = 19.4°, which corresponds to the 100 strong reflections of CS, is also present. Finally, for the CS-TiO_2_-GO-GLA (Figure 4D) nanocomposite the peak for CS was evident at 20.0°, while for the TiO_2_ peaks they appeared at 25.0°, 38.0°, 47.8°,48.1°, 54.4°, 63.1°, and for GO the peak at 13.0° corresponding to the presence of well-dispersed GO sheets.

The crystallinity index (Xc) was calculated to understand better the crystallinity of the beads (Table 1). Although the original methodology was reported for starch, eventually, it was adjusted for other polymers like CS [68]. The results indicate that the addition of one of the nanomaterials increased the crystallinity of the beads, a finding that agrees with higher thermal and mechanical resistance observed. This effect has been reported with the use of other nanofillers such as nanoclays, carbon nanotubes, and graphene oxide [69,70,71].

If the crystallinity is increasing with the introduction of the nanomaterials, it means that nanomaterials are well dispersed between the polymer matrix, especially for TiO_2_, which has a higher crystallinity as compared to CS beads, probably due to the intermolecular hydrogen bonding, between CS and the nanomaterial giving a relatively ordered adjustment of the polymer chains along the GO nanosheets or TiO_2_ nanoparticles [70]. The introduction of TiO_2_ leads to a higher crystallinity index than with GO, probably due to the fact that TiO_2_, having high crystallinity, might be more effective in achieving superior reinforcement for composite materials [72]. However, the presence of both nanomaterials decreased crystallinity compared to CS-TiO_2_-GLA beads due to the lower crystallinity of the GO. More research is needed to understand why the two nanomaterials are not improving the crystallinity together and the thermal behavior, which could be due to the 3D morphology of the TiO_2_ and the 2D morphology of GO nanosheets in the tridimensional matrix of chitosan, decreasing the chitosan bindings.

#### 2.3.3. Scanning Electron Microscopy (SEM)

**Figure 5:**
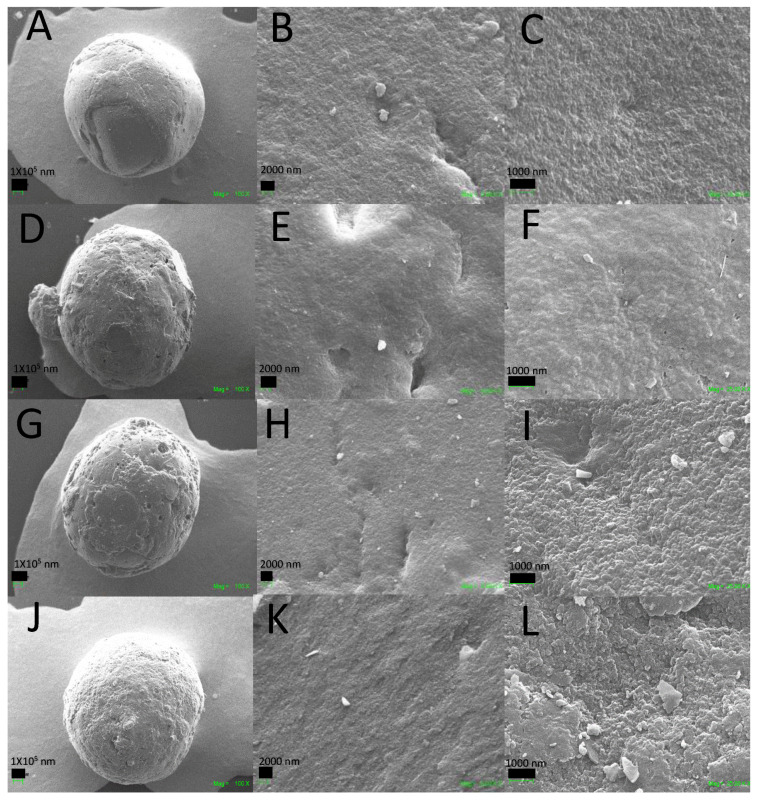
SEM images. Morphology of the beads: CS-GLA (**A**) at 100×, (**B**) at 1000×, (**C**) at 25,000×; CS-GO-GLA (**D**) at 100×, (**E**) at 1000×, (**F**) at 25000×; CS-TiO_2_-GLA (**G**) at 100×, (**H**) at 1000×, (**I**) at 25,000×; CS-TiO_2_-GO-GLA (**J**) at 100×, (**K**) at 1000×, (**L**) at 25,000×.

The microstructure of the CS composite beads was studied by the SEM technique. Figure 5 shows the surface analysis of the different composite beads. Figure 5A–C display a smooth surface for the CS-GLA crosslinking beads, mainly due to the chemical crosslinking and hydrogen bindings [73]. By adding GO and TiO_2_ to the CS beads, the nanomaterial increases the roughness due to their texture and loss of the semi-crystalline chitosan structure [70]. Figure 5D–L illustrate the surface texture and porosity of the CS-GO-GLA and CS-GO-TiO_2_-GLA beads with holes on the surface that support nutrient transportation and provide a waste outlet for cells [73]. Solvent evaporation and polysaccharide chain separation during the bead formation are the main reasons for the porous structure [74].

**Figure 6:**
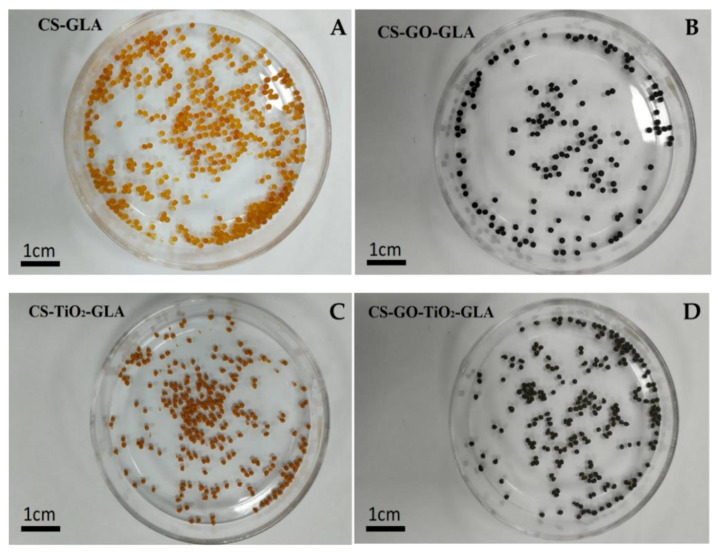
Images of the different bead composites: (**A**) CS-GLA, (**B**) CS-GO-GLA, (**C**) CS-TiO_2_-GLA, (**D**) CS-GO-TiO_2_-GLA.

The color of the CS-GLA beds ranged from yellow to light-red (Figure 6) due to the imine -C=N bonds formed between the -NH_2_ and -CHO groups [56]. However, with the introduction of GO and TiO_2_ in the beads, there was an obvious darkening effect [75].

#### 2.3.4. Thermogravimetric Analysis (TGA) and Differential Scanning Calorimetry (DSC) Analysis

The thermogravimetric analysis, with four decomposition steps, is shown in Figure S1. Between 25°C and 100°C water evaporation mainly occurs and small fragments of the periphery groups of the chitosan chains are broken off. After 200 °C, degradation of chitosan occurs for all the formulations, with a rapid weight loss centered at 250 °C according to the derivative thermogravimetric (DTGA) curves. The final stage, due to the pyrolysis of the chitosan polymer, occurs in the range between 300 °C and 470 °C [70]. However, incorporation of TiO_2_ into the CS-GLA beads increased the thermal resistance, as observed in Figure S1, showing an increase in the temperature degradation for each stage and a higher remaining material after 600 °C, which indicates a higher thermal resistance of the beads. 

The decomposition temperatures at 3% (Td_3%_, 3% of mass loss) for all the composites are listed in Table 2. The Td_3%_ for CS-GLA shows higher thermal stability compared to pristine CS due to the crosslinking reactions. The Td_3%_ dramatically increased with GO incorporation, demonstrating thermal stabilization with the nanofiller introduction, possible due to the higher crystallinity of the nanomaterial. From this table, we can conclude that the beads will not be thermally affected by body conditions (37 °C) since they are thermally degraded only between 215 °C and 500 °C. 

#### 2.3.5. TEM Images of the Nanocomposite Beads 

**Figure 7:**
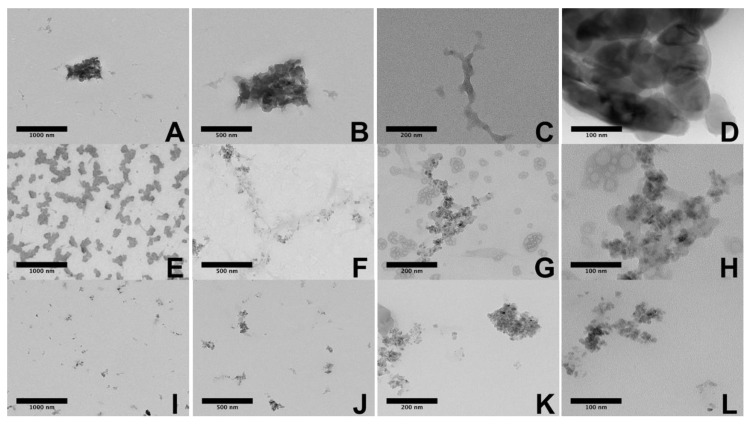
HR-TEM images of the different beads: CS-GO-GLA (**A**) 1000 nm, (**B**) 500 nm, (**C**) 200 nm, (**D**) 100 nm; CS-GO-TiO_2_-GLA (**E**) 1000 nm, (**F**) 500 nm, (**G**) 200 nm, (**H**) 100 nm; CS-TiO_2_-GLA (**I**) 1000 nm (**J**) 500 nm (**K**) 200 nm, (**L**) 100 nm.

TEM images of the CS nanocomposite beads are shown in Figure 7. The nanoparticles of TiO_2_ and nanosheets of GO are well distributed in the polymer matrix (Figure 7A–C), confirming that the proposed is suitable for producing well-dispersed compatible composites of GO and TiO_2_ with CS, probably by hydrogen bonding and hydrophobic interactions that modify the surface tension of the nanomaterials [51]. Figure 7D shows the presence and distribution of the GO sheets well distributed in the chitosan matrix (CS-GO-GLA beads). Despite the low amount introduced in the composite the presence of TiO_2_ nanoparticles in the polymer matrix is shown in Figure 7E–L. The behavior in the TEM analysis is like that reported by Li et al. [76]. They found that the TiO_2_ was loaded on graphene sheets, with apparent accumulation along the matrix, caused by the presence of carboxylic groups on the graphene oxide as displayed in the Figure 7H. However, distinguishing between GO and TiO_2_ was harder in the CS-GO-TiO_2_-GLA TEM images. 

#### 2.3.6. In Vivo Biomodel Tests 

**Figure 8:**
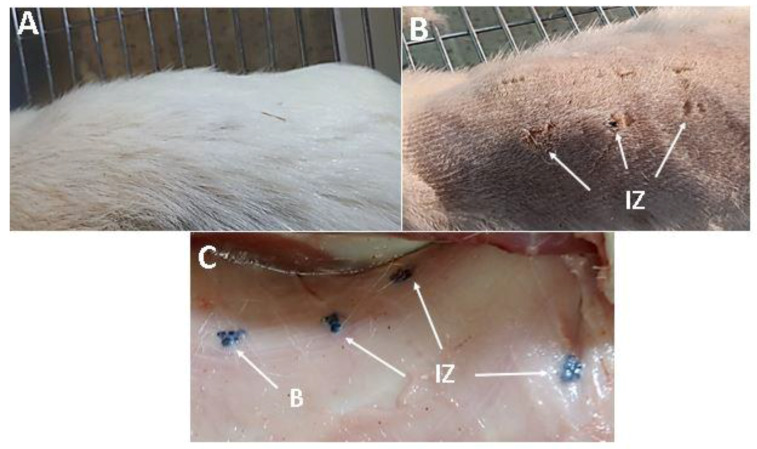
Zone of subdermal implantation in Wistar rats after 30, 60, and 90 days (**A**) Hair recovery, (**B**) Absence of immune responses, (**C**) Subdermal implantation zones with samples encapsulated by scar tissue. **B:** Beads. **IZ:** Implantation zone.

Figure 8 shows the macroscopic appearance during the recovery of the samples. Average hair growth indicates a normal healing process (Figure 8A). Figure 8B shows the implantation area after the application of the trichotomy. Dry skin and healing with youthful appearance and persistence of stitches support a complete normal healing process. Figure 8C corresponds to subdermal tissue, where implanted beads (B) are included in the tissue and surrounded by a layer of transparent soft tissue.

**Figure 9:**
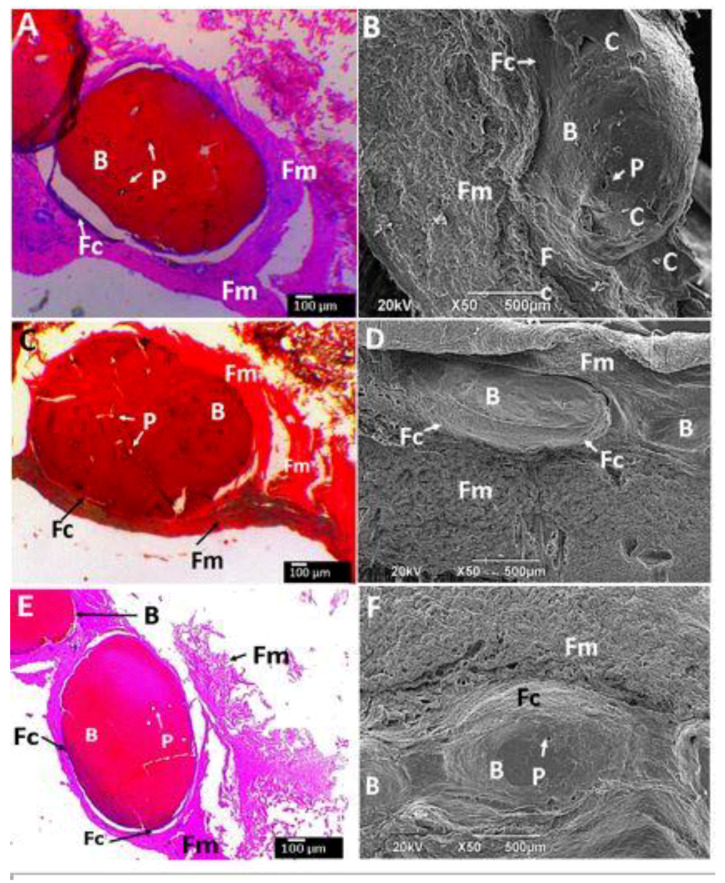
Beads implanted in subdermal tissue. (**A**,**B**): CS-GO-TiO_2_-GLA. (**C**,**D**): CS-TiO_2_-GLA. (**E**,**F**): CS-TiO_2_-GLA. (**A**,**C**,**E**): Hematoxylin and eosin stain technique at 4×. (**B**,**D**,**F**): SEM technique. B: Bead. P: Pore. Fc: Fibrous capsule. Fm: Fibrous membrane. C: Cell.

**Figure 10:**
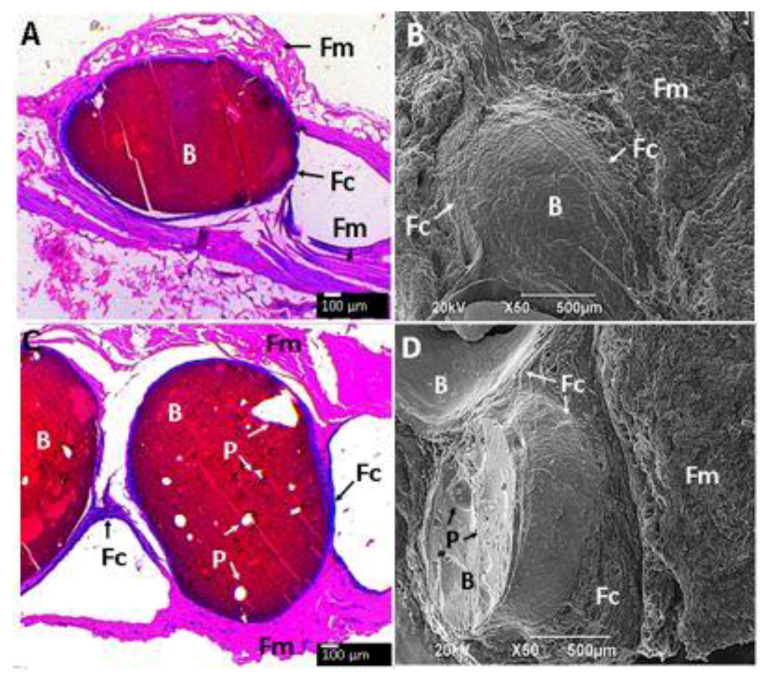
Beads embedded in subdermal tissue. (**A**,**B**): CS-GO-GLA. (**C**,**D**): CS-GO-TiO_2_-GLA. (**A**,**C**): Hematoxylin and eosin (H-E) stain technique to 4×. (**B**,**D**): SEM technique. B: Bead. P: Pore. Fc: Fibrous capsule. Fm: Fibrous membrane.

The subdermal area of the Wistar rats is shown in Figure 9A–F and Figure 10A–D show histological and SEM images of the beads after implantation for 90 days. All the CS composite beads were surrounded by soft fibrous tissue (Fm). However interestingly, each bead was surrounded by a material of fibrous nature different from the fibrous membrane capsule (Fc) that surrounded the entire beads (Figure 9). The H&E stain technique allows one to observe a dark-blue zone (Fc) corresponding to the different fibrous tissue seen in direct contact with the beads (Figure 9A,C,E). At the same time, the SEM technique also corroborates the diverse nature of that tissue by a homogeneous and smooth fibrous (Fc) appearance, far different from the rough tissue (Fm). 

It has been demonstrated with in vivo evaluations that chitosan suffers enzymatic degradation [77,78]. The rate and ratio of the degradation process depend on the degree of deacetylation of the chitosan, molecular weight, crosslinking reaction, interaction with other components, and nanomaterials, among others.

Chitosan demonstrates capacity for cell adhesion and biodegradability. For example, Fujita et al. [79] reported chitosan-based hydrogels with the ability to degrade after 20 days under sub-dermal implantation conditions [79]. Vaishali et al. [80] observed chitosan-collagen sponge degradation under subdermal implantation conditions in Wistar rats after 42 days without adverse immune system responses [80]. 

**Figure 11:**
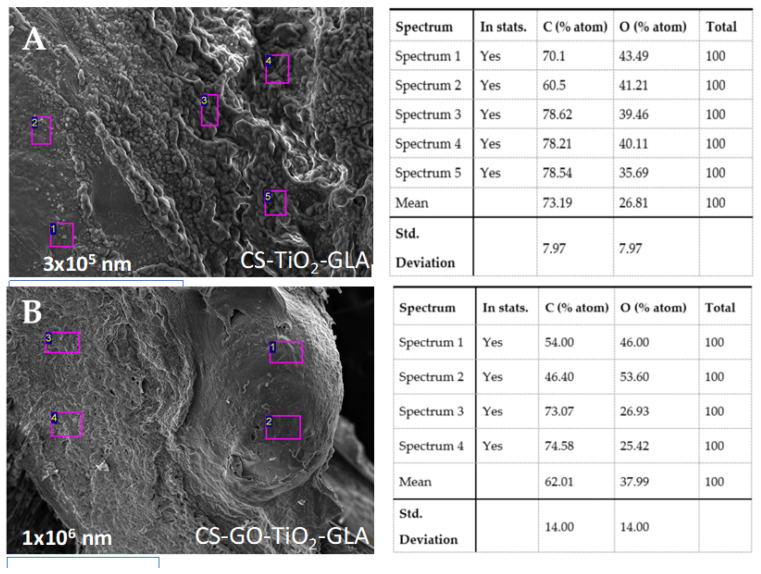
Beads implanted in subdermal tissue. (**A**) CS-TiO_2_-GLA. (**B**) CS-GO-TiO_2_-GLA. SEM-EDS technique.

No allergenic reactions were observed in Wistar rats with the nanocomposites. Figure 11 shows the SEM-EDS results for A: CS-TiO_2_-GLA and B: CS-GO-TiO_2_-GLA after 90 days of implantation. Carbon and oxygen presence are evident surrounding the beads demonstrating the growth of tissues without the presence of pus. 

The presence of carbon and oxygen by themselves are not indicators of biocompatibility since they can be found in all tissues. However, the biocompatibility of implanted materials was evidenced macroscopically through the scarring process with the healthy growth of new hair. 

Microscopically, the biocompatibility was observed with particles surrounded by a fibrous collagen capsule beginning a resorption process with the presence of pores in the different implanted particles. Low degradation of the structure confirms the stability of the beads, which could be advantageous for long-term applications.

### 2.4. Analysis of Porosity Results

A morphometric study from the 10× images was conducted to determine the percentage of resorption of the beads. Resorption percentages are presented in Table S1. The five formulations were very stable, showing little resorption after three months of implantation. For the CS-GLA formulation, low reabsorption percentage (0.17%) indicates the stability of the material. When GO is incorporated, the content stabilizes due to the nanofiller effect, and the porosity decreases to 0.006%. When TiO_2_ is added to the CS-GLA beads, the percentage of porosity increased almost twice. However, if the GO is also incorporated, the resorption increased by 840% (the highest rate obtained), which could be explained by the higher compatibility shown by the TiO_2_ nanoparticles, which would favor the resorption process.

It is also striking that the CS-GLA formulation showed the presence of skin histiocytes or macrophages, probably due to the presence of the CS producing a robust inflammatory response. However, when the GO and TiO_2_ are incorporated, the answer appears to be healthy for a typical foreign body reaction, possibly because the nanomaterials stabilize the CS by chemical interactions.

**Figure 12:**
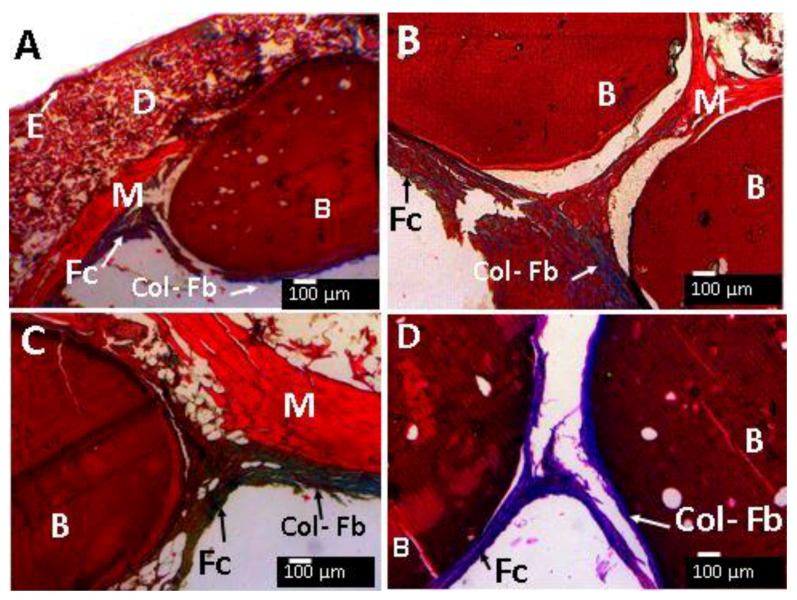
Beads implanted in subdermal tissue. (**A**) CS-GO-GLA. (**B**) CS-TiO_2_- GLA. (**C**) CS-GO-GLA. (**D**) CS-GO-TiO_2_-GLA. (**A**) at 4×. (**B**), (**C**), and (**D**) at 10×. E: Epidermis. D: Dermis. M: Muscle. B: Bead. Fc: Fibrous capsule. COL - Fb: Type I collagen fiber. Masson′s trichrome stain (MT) technique.

From the H&E technique is not possible to directly determine the nature of the Fc tissue. However, Masson′s Trichromacy technique (MT) indicated that the fibrous capsule surrounding each bead corresponded to collagen type I because of the blue appearance after staining (Figure 12A–D).

**Figure 13:**
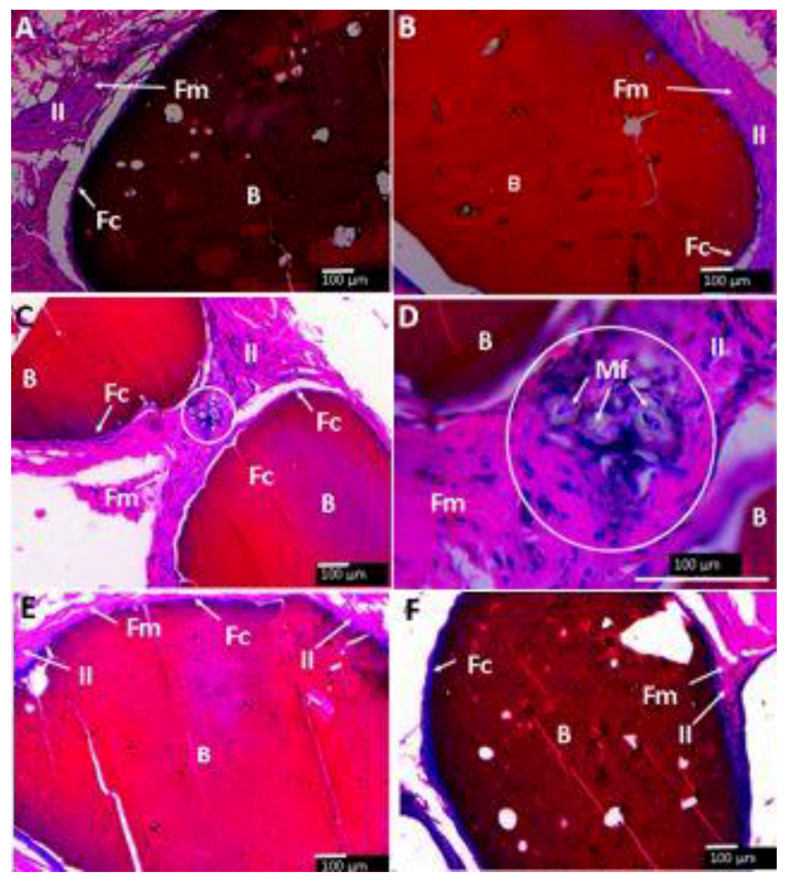
Beads implanted in subdermal tissue. (**A**) CS-GO-TiO_2_-GLA. (**B**) CS-TiO_2_- GLA. (**C**,**D**) CS-GLA. (**E**) CS-GO-GLA. (**F**) CS-GO-TiO_2_-GLA. II: Inflammatory infiltrate. Fm. Fibrous membrane. Fc: Fibrous capsule. B: Bead. Mf: Macrophages. 10× images. Masson′s trichrome stain (MT) technique.

When examining the images at higher magnification, all the beads are surrounded by an inflammatory infiltrate (II). Figure 13C,D show the presence of a group of macrophage/histiocyte compatible cells (circle area), a good indication for a healthy healing process.

In general, all formulations (Figure 13A–F) have a fibrous encapsulation, which is normal when biomaterials are implanted in the subdermal tissue area. Besides that, each bead was surrounded by a capsule consisting of type I collagen. The inflammatory response was similar for all samples, with very identical histological appearance, except the sample of CS-GLA, which had a presence of macrophage/histiocyte cells. 

## 3. Materials and Methods 

Titanium isopropoxide (TTIP; reagent grade, 99%, Aldrich, (Palo Alto, CA, USA), 2-propanol, nitric acid (HNO_3_), and distilled water were used for TiO_2_ nanoparticles synthesis. Graphene oxide was synthesized using graphite (325 mesh, Alfa-Aesar, Tewksbury, MA, USA), sulfuric acid (H_2_SO_4_), potassium permanganate (KMnO_4_), hydrogen peroxide (H_2_O_2_), and 2-propanol (Merck, Burlington, MA, USA). CS beads were prepared using chitosan (from shrimp shells) with a molecular weight of Mv 144.000, measured by capillary viscometry (using an Ubbelohde 0C viscometer, Schott, Barcelona, Spain). *K* and *a* constants for CS in the solvent used (acetic acid 0.3 M + sodium acetate 0.2 M) at 25 °C are 0.074 mL/g and 0.76, respectively [81], for the calculation of the molecular weight with the Mark-Houwink-Sakurada equation (Equation (1)):

(1) $$\left[\eta \right]=K{\left(Mv\right)}^{a}$$

The degree of deacetylation determined by ^1^H-NMR using a 400 MHz AVANCE II spectrometer (Bruker, Billerica, MA, US) at a temperature of 300 K and elemental analysis of the CS was 89%. The sample was dissolved in D_2_O with two drops of trifluoroacetic and 3-(trimethylsilyl)propionic acid-d_4_ as reference salt. The elemental analysis was performed using a Flash EA 1112 system (Thermo Electron, Waltham, MA, US). 

### 3.1. Synthesis of TiO_2_ Nanoparticles 

TiO_2_ nanospheres were synthesized using a previously reported method [51,67]. Briefly, 15 mL of TTIP were mixed with 15 mL (0.016 mol) of 2-propanol (precursor solution) [51]. Then, 250 mL of distilled water at pH 2 (3 M HNO_3_) was prepared, while the precursor solution was added dropwise with vigorous stirring. After that, the hydrolysis of TTIP was produced at 60 °C and 20 h of reaction. After solvent evaporation, a yellow crystal precipitated. A yellow-white powder was obtained after ethanol washing and drying at 100 °C. The final step consisted of solid calcination at 400 °C for two hours. 

#### Characterization of TiO_2_ Nanoparticles

TiO_2_ nanoparticle synthesis and characterization were already reported [51]. TiO_2_ nanoparticle morphology was studied by TEM (ARM 200 F, JEOL, Tokyo, Japan) at 20 kV. Samples for TEM measurements were prepared by placing a drop of TiO_2_ on a carbon-coated standard copper grid (400 mesh) and evaporating the solvent. On the other hand, X-ray diffractometry (XRD) experiments for TiO_2_ were performed using a PANalytical X′Pert PRO diffractometer (Malvern Panalytical, Jarman Way, Royston, UK), with Cu Kα1 radiation (1.540598 Å) and Kα2 (1.544426 Å), in a 2θ range between 5° and 80°. FTIR experiments on TiO_2_ were performed in attenuated total reflectance mode using a diamond tip accessory (instrument model, Shimadzu, Kyoto, Japan).

### 3.2. Preparation of Graphene Oxide (GO) 

GO was prepared by the modified Hummers method [33,82,83,84,85]. 

#### Characterization of Graphene Oxide (GO) 

X-ray diffractometry (XRD) experiments for GO was performed in a PANalytical X′Pert PRO diffractometer (Malvern Panalytical), using Cu Kα1 radiation (1.540598 Å) and Kα2 (1.544426 Å), in a 2θ range between 5° and 50°. Bragg′s law (Equation (2)) was used to calculate the interlayer distance in the GO:

(2) $$d=\frac{\lambda }{2sen\theta }$$

where *d* is the lattice spacing, *λ* is the X-ray wavelength, and *θ* is the angle of incidence.

The Raman spectrum recorded with an Invia Raman Microscope in a wavelength laser of 514.5 nm (Renishaw, New Mills, Gloucestershire, UK) was used for GO characterization. Atomic force microscopy (AFM) experiments were performed in tapping mode using a Multimode AFM (Veeco, Plainview, NY, US) equipped with a Nanoscope Iva control system (software version 6.14r1). Silicon tapping probes (RTESP, Veeco, Plainview, NY, US) were used with a resonance frequency of ~300 kHz, scan rates of 0.4 Hz, 5 × 5, 2 × 2 μm^2^. AFM images were taken for each sample. Topography was examined by topographical AFM mode.

### 3.3. Synthesis of CS Nanocomposite Beads 

For the preparation of chitosan/graphene oxide beads and glutaraldehyde (CS-GO-GLA), 2 g of chitosan was dissolved in 100 mL of acetic acid (1% *v/v*). Then, GO was added to this solution (10 mg) and stirred until a homogeneous dispersion was obtained. The procedure for obtaining TiO_2_ beads was similar, but 100 mg of TiO_2_ was added instead of GO (CS-TiO_2_-GLA). The above process was repeated, and 100 mg of TiO_2_ and 10 mg of GO were added for the chitosan/graphene oxide/TiO_2_ beads (CS-GO-TiO_2_-GLA) and stirred until a homogeneous dispersion was obtained.

The formation of the different nanocomposite beads was carried out using the phase inversion technique [86], with a solution of sodium hydroxide (NaOH) 0.5 M. Each type of mixture prepared up to this point was titrated with the NaOH solution, which allowed the formation of insoluble spheres. The spheres formed were left under stirring in the primary solution for a period of 4 h. Subsequently, they were washed with abundant distilled water until neutral pH and allowed to air dry for approximately 24 h until constant dry weight. Chitosan nanocomposite beads (CS-GLA, CS-GO-GLA, CS-TiO_2_-GLA, and CS-GO-TiO_2_-GLA) were obtained by dispersing the beads obtained previously in a 2.0% *v/v* solution of GLA in water (CS: GLA 1:1 molar ratio), under gentle stirring for 24 h at ambient temperature. Subsequently, they were washed with abundant distilled water to remove excess GLA and finally air-dry until constant dry weight and placed in a desiccator at 10% relative humidity (RH) until the time of the test [87]. 

#### Nanocomposite Beads Characterization

XRD experiments were run with the same procedure and apparatus than the previously reported range of 2θ from 0–80°, and time per step 304.390 s. According to Nara–Komiya methodology [88], the percentage of crystallinity from the XRD technique (Xc%) was calculated using Equation (3):

(3) $$X_{c}\left(\%\right)=\left(\frac{A_{c}}{A_{T}}\right)\times 100$$

where A_C_ is the area under the peaks that represents the crystalline region, and A_T_ is the total area of the crystalline and amorphous region.

Thermogravimetric analysis was run on a Netzsch TG Libra 209 instrument (TA Instruments, New Castle, DE, USA) in a temperature range between 30–700 ± 2 °C. The melting temperature was determined by differential scanning calorimetry using a DSC2A-00181 system (TA Instruments) at 10 °C/min.

FTIR analysis of the beads was performed in ATR mode (attenuated total reflectance) with a diamond tip on an IR Affinity-1 spectrometer (Shimadzu, Kyoto, Japan). SEM analysis was performed on a scanning electron microscope (JSM-6490LA, JEOL) using an acceleration voltage of 20 kV, in which the samples were coated with a copper bath. The samples for SEM- energy-dispersive spectroscopy (EDS) were fixed in 75% alcohol for 48 h and dehydrated using an ascending alcohol gradient (70%, 80%, 95%, and 100%), after which they adhered on carbon tape. Then, an exterior gold coating was applied (Model Desk IV equipment, Denton Vacuum, Moorestown, NJ, USA) to generate a conductive surface. Subsequently, a JEOL Model JSM 6490 LV microscope was used to inspect the samples in the secondary electron mode with an acceleration voltage of 20 kV to obtain electron microscope images. Additionally, chemical microanalysis was carried out on several inspection areas, employing the energy-dispersive spectroscopy (EDS) probe of a Model INCAPentaFETx3 instrument (Oxford Instruments, Abingdon, UK). The EDS probe which had a resolution of 137 eV to 5.9 keV, while SEM had a resolution of 15 nm with an acceleration voltage of 1 kV and a working distance of 6 mm in the secondary electron mode. 

The morphology of the GO and TiO_2_ nanoparticles and their dispersion in the composite beads were analyzed by TEM (JEOL ARM 200 F) operating at 20 kV. Samples for TEM measurements were prepared by placing a drop of TiO_2_ on a carbon-coated standard copper grid (400 mesh) and evaporating the solvent.

### 3.4. In Vivo Biomodel Tests

Samples of 9.7 mg of the different types of beads were implanted in subdermal preparations of three adult male Wistar rats supplied and hosted by the LABBIO laboratory of the Universidad del Valle (Cali, Colombia). Biomodels were sedated by the intramuscular application of ketamine 70 mg/kg and xylazine 30 mg/kg (Holliday Scott S Laboratory, Buenos Aires, Argentina) to prepare the samples. Each preparation consisted of an incision one cm long and three cm deep on the dorsal surface of the biomodels. After 90 days of implantation the biomodel animals were euthanized via intraperitoneal injection of sodium pentobarbital 390 mg/mL and 50 mg/mL of sodium diphenylhydantoin (Euthanex^®^, INVENT Laboratory, Santiago de Cali, Colombia) at a dose of 100 mg/kg. The samples were processed for histological analysis by the hematoxylin and eosin (H-E) and Masson′s trichrome stain (MT) techniques.

The images obtained were processed using the ImageJ 1.3 image analysis program (National Institutes of Health, Bethesda, MD, USA), which was calibrated before measuring with the scale that was in each image. From this information, the resorption percentages of the areas corresponding to the pores were determined by applying the following equation:

Resorption percentage = Resorbed area × 100/Total area of the sample(4)

This research was reviewed, supported, and supervised by the Institutional Ethics Review Committee with experimental animals of the Universidad del Valle (CEAS 012-019).

### 3.5. Statistical Analysis

In vivo studies of the beads are presented as the mean value of at least three replicates ± SD. The Statgraphics Centurion XVI program (Statgraphics, The Plains, VA, USA) was used for these statistical analyses. 

## 4. Conclusions

The successful preparation of very stable and biocompatible CS beads based on TiO_2_ nanoparticles and GO nanosheets was evident from chemical, thermal, and biological tests. The chemical crosslinking of the CS using glutaraldehyde increased the decomposition temperature due to the increased crystallinity, especially for CS-GLA-GO-TiO_2_ beads. However, the introduction of the nanoparticles increased the thermal stability, as evidenced by TGA and DTGA results. The thermal results correlated with the crystallinity indexes and in vivo subdermal implantation studies during 90 days in Wistar rats, where remaining material was found after 90 days without the presence of pus or an immune response due to the high stability of the material and the reinforcing effect of the nanofillers. Graphene oxide and titanium dioxide nanoparticles were efficiently distributed in the polymer matrix as TEM results showed. The morphology of the beads become rougher with the introduction of the nanoparticles. The high balance between long-term stability, porosity, and surface irregularity observed by SEM could be useful for long-term applications, for example, bone tissue regeneration, where porosity and surface irregularities are useful for cell-adhesion and proliferation. Finally, examination of the beads using staining by the Masson′s Trichrome and hematoxylin and eosin (H-E) staining techniques after the in vivo studies showed collagen type I as the main constituent of the surrounding fibrous capsule on the surface of the beads, indicating the strong biocompatibility of the material. Very interestingly, CS-GLA beads displayed the presence of macrophage/histiocyte compatible cells, which demonstrates the beginning of a phagocytic process of the content. 

## Figures and Tables

**Table 1:** The crystallinity index (Xc) of the different formulations calculated from the XRD analysis.

Formulation	Xc (%)
CS-GLA	10.8
CS-GO-GLA	15.5
CS-TiO_2_-GLA	24.0
CS-TiO_2_-GO-GLA	21.8

**Table 2:** Td_3%_ of the nanocomposite beads (CS-GLA, CS-GO-GLA, CS-TiO_2_-GLA, and CS-TiO_2_-GO-GLA).

Sample	Td_3%_ (°C)
CS-GLA	92.8
CS-GO-GLA	131.3
CS-TiO_2_-GLA	85.8
CS-TiO_2_-GO-GLA	124.7
CS pure	44.2

Td_3%_: decomposition temperature at 3% weight loss, Tg: glass transition temperature.

## Supplementary Materials

The supplementary materials are available online.

## References

[1] Khorshidi S., Solouk A., Mirzadeh H., Mazinani S., Lagaron J.M., Sharifi S., Ramakrishna S. (2016). A review of key challenges of electrospun scaffolds for tissue-engineering applications. J. Tissue Eng. Regen. Med..

[2] Gomes M.E., Azevedo H.S., Moreira A.R., Ellä V., Kellomäki M., Reis R.L. (2008). Starch-poly (ε-caprolactone) and starch-poly (lactic acid) fibre-mesh scaffolds for bone tissue engineering applications: Structure, mechanical properties and degradation behaviour. J. Tissue Eng. Regen. Med..

[3] Hollister S.J. (2005). Porous scaffold design for tissue engineering. Nat. Mater..

[4] Antunes J.C., Oliveira J.M., Reis R.L., Soria J.M., Gómez--Ribelles J.L., Mano J.F. (2010). Novel poly (L-lactic acid)/hyaluronic acid macroporous hybrid scaffolds: Characterization and assessment of cytotoxicity. J. Biomed. Mater. Res. Part A.

[5] Stratton S., Shelke N.B., Hoshino K., Rudraiah S., Kumbar S.G. (2016). Bioactive polymeric scaffolds for tissue engineering. Bioact. Mater..

[6] Dhandayuthapani B., Yoshida Y., Maekawa T., Kumar D.S. (2011). Polymeric scaffolds in tissue engineering application: A review. Int. J. Polym. Sci..

[7] Muzzarelli A.A.R., Mehtedi E.M., Mattioli-Belmonte M. (2014). Emerging Biomedical Applications of Nano-Chitins and Nano-Chitosans Obtained via Advanced Eco-Friendly Technologies from Marine Resources. Mar. Drugs.

[8] Seol Y.-J., Lee J.-Y., Park Y.-J., Lee Y.-M., Rhyu I.-C., Lee S.-J., Han S.-B., Chung C.-P. (2004). Chitosan sponges as tissue engineering scaffolds for bone formation. Biotechnol. Lett..

[9] Şenel S., McClure S.J. (2004). Potential applications of chitosan in veterinary medicine. Adv. Drug Deliv. Rev..

[10] Di Martino A., Sittinger M., Risbud M.V. (2005). Chitosan: A versatile biopolymer for orthopaedic tissue-engineering. Biomaterials.

[11] Aranaz I., Mengíbar M., Harris R., Paños I., Miralles B., Acosta N., Galed G., Heras Á. (2009). Functional characterization of chitin and chitosan. Curr. Chem. Biol..

[12] Archana D., Dutta J., Dutta P.K. (2013). Evaluation of chitosan nano dressing for wound healing: Characterization, in vitro and in vivo studies. Int. J. Biol. Macromol..

[13] Bui V.K.H., Park D., Lee Y.-C. (2017). Chitosan combined with ZnO, TiO_2_ and Ag nanoparticles for antimicrobial wound healing applications: A mini review of the research trends. Polymers.

[14] Rizeq B.R., Younes N.N., Rasool K., Nasrallah G.K. (2019). Synthesis, Bioapplications, and Toxicity Evaluation of Chitosan-Based Nanoparticles. Int. J. Mol. Sci..

[15] Mansoori G.A. (2005). Principles of Nanotechnology: Molecular-Based Study of Condensed Matter in Small Systems.

[16] Saji V.S., Choe H.C., Yeung K.W.K. (2010). Nanotechnology in biomedical applications: A review. Int. J. Nano Biomater..

[17] Tamayo Marín A.J., Londoño R.S., Delgado J., Navia Porras P.D., Valencia Zapata E.M., Mina Hernandez H.J., Valencia H.C., Grande Tovar D.C. (2019). Biocompatible and Antimicrobial Electrospun Membranes Based on Nanocomposites of Chitosan/Poly (Vinyl Alcohol)/Graphene Oxide. Int. J. Mol. Sci..

[18] López Tenorio D., Valencia H.C., Valencia C., Zuluaga F., Valencia E.M., Mina H.J., Grande Tovar D.C. (2019). Evaluation of the Biocompatibility of CS-Graphene Oxide Compounds In Vivo. Int. J. Mol. Sci..

[19] Grande Tovar C.D., Castro J.I., Valencia C.H., Navia Porras D.P., Hernandez M., Herminsul J., Valencia M.E., Velásquez J.D., Chaur M.N. (2019). Preparation of Chitosan/Poly (Vinyl Alcohol) Nanocomposite Films Incorporated with Oxidized Carbon Nano-Onions (Multi-Layer Fullerenes) for Tissue-Engineering Applications. Biomolecules.

[20] Alireza K., Ali M.G. (2011). Nanostructured Titanium Dioxide Materials: Properties, Preparation and Applications.

[21] Gerhardt L.-C., Jell G.M.R., Boccaccini A.R. (2007). Titanium dioxide (TiO_2_) nanoparticles filled poly (D, L lactid acid)(PDLLA) matrix composites for bone tissue engineering. J. Mater. Sci. Mater. Med..

[22] Marszewski M., Jaroniec M. (2015). Scaffold-assisted synthesis of crystalline mesoporous titania materials. RSC Adv..

[23] Yin Z.F., Wu L., Yang H.G., Su Y.H. (2013). Recent progress in biomedical applications of titanium dioxide. Phys. Chem. Chem. Phys..

[24] Feng B., Weng J., Yang B.C., Qu S.X., Zhang X.D. (2003). Characterization of surface oxide films on titanium and adhesion of osteoblast. Biomaterials.

[25] Hanawa T. (2011). A comprehensive review of techniques for biofunctionalization of titanium. J. Periodontal Implant. Sci..

[26] Mousavi S.M., Hashemi S.A., Ghasemi Y., Amani A.M., Babapoor A., Arjmand O. (2019). Applications of graphene oxide in case of nanomedicines and nanocarriers for biomolecules: Review study. Drug Metab. Rev..

[27] Kuila T., Bose S., Mishra A.K., Khanra P., Kim N.H., Lee J.H. (2012). Chemical functionalization of graphene and its applications. Prog. Mater. Sci..

[28] Song Y., Wei W., Qu X. (2011). Colorimetric biosensing using smart materials. Adv. Mater..

[29] Tao Y., Lin Y., Huang Z., Ren J., Qu X. (2013). Incorporating graphene oxide and gold nanoclusters: A synergistic catalyst with surprisingly high peroxidase--like activity over a broad pH range and its application for cancer cell detection. Adv. Mater..

[30] Mohanty N., Berry V. (2008). Graphene-based single-bacterium resolution biodevice and DNA transistor: Interfacing graphene derivatives with nanoscale and microscale biocomponents. Nano Lett..

[31] Kelly K.F., Billups W.E. (2012). Synthesis of soluble graphite and graphene. Acc. Chem. Res..

[32] Liu X., Ma R., Wang X., Ma Y., Yang Y., Zhuang L., Zhang S., Jehan R., Chen J., Wang X. (2019). Graphene oxide-based materials for efficient removal of heavy metal ions from aqueous solution: A review. Environ. Pollut..

[33] Valencia C., Valencia C., Zuluaga F., Valencia M., Mina J., Grande-Tovar C. (2018). Synthesis and Application of Scaffolds of Chitosan-Graphene Oxide by the Freeze-Drying Method for Tissue Regeneration. Molecules.

[34] Suk J.W., Piner R.D., An J., Ruoff R.S. (2010). Mechanical properties of monolayer graphene oxide. ACS Nano.

[35] Li D., Müller M.B., Gilje S., Kaner R.B., Wallace G.G. (2008). Processable aqueous dispersions of graphene nanosheets. Nat. Nanotechnol..

[36] Sanchez V.C., Jachak A., Hurt R.H., Kane A.B. (2012). Biological interactions of graphene-family nanomaterials: An interdisciplinary review. Chem. Res. Toxicol.

[37] Kiew S.F., Kiew L.V., Lee H.B., Imae T., Chung L.Y. (2016). Assessing biocompatibility of graphene oxide-based nanocarriers: A review. J. Control. Release.

[38] Pan Y., Sahoo N.G., Li L. (2012). The application of graphene oxide in drug delivery. Expert Opin. Drug Deliv..

[39] Ji H., Sun H., Qu X. (2016). Antibacterial applications of graphene-based nanomaterials: Recent achievements and challenges. Adv. Drug Deliv. Rev..

[40] Xu Y., Zeng J., Chen W., Jin R., Li B., Pan Z. (2018). A holistic review of cement composites reinforced with graphene oxide. Constr. Build. Mater..

[41] Li Y., Wu Q., Zhao Y., Bai Y., Chen P., Xia T., Wang D. (2014). Response of microRNAs to in vitro treatment with graphene oxide. ACS Nano.

[42] Liao K.-H., Lin Y.-S., Macosko C.W., Haynes C.L. (2011). Cytotoxicity of graphene oxide and graphene in human erythrocytes and skin fibroblasts. ACS Appl. Mater. Interfaces.

[43] Chang Y., Yang S.-T., Liu J.-H., Dong E., Wang Y., Cao A., Liu Y., Wang H. (2011). In vitro toxicity evaluation of graphene oxide on A549 cells. Toxicol. Lett..

[44] Sharma C., Dinda A.K., Potdar P.D., Chou C.-F., Mishra N.C. (2016). Fabrication and characterization of novel nano-biocomposite scaffold of chitosan-gelatin-alginate-hydroxyapatite for bone tissue engineering. Mater. Sci. Eng. C.

[45] Durkut S., Elçin Y.M., Elçin A.E. (2006). Biodegradation of chitosan-tripolyphosphate beads: In vitro and in vivo studies. Artif. Cells Blood Sub..

[46] Jayakumar R., Menon D., Manzoor K., Nair S.V., Tamura H. (2010). Biomedical applications of chitin and chitosan based nanomaterials—A short review. Carbohydr. Polym..

[47] Lim S.M., Song D.K., Oh S.H., Lee-Yoon D.S., Bae E.H., Lee J.H. (2008). In vitro and in vivo degradation behavior of acetylated chitosan porous beads. J. Biomater. Sci. Polym. Ed..

[48] Kim S.E., Park J.H., Cho Y.W., Chung H., Jeong S.Y., Lee E.B., Kwon I.C. (2003). Porous chitosan scaffold containing microspheres loaded with transforming growth factor-β1: Implications for cartilage tissue engineering. J. Control. Release.

[49] Kravanja G., Primožič M., Knez Ž., Leitgeb M. (2019). Chitosan-Based (Nano)Materials for Novel Biomedical Applications. Molecules.

[50] Mohandas A., Deepthi S., Biswas R., Jayakumar R. (2018). Chitosan based metallic nanocomposite scaffolds as antimicrobial wound dressings. Bioact. Mater..

[51] Zapata P.A., Palza H., Delgado K., Rabagliati F.M. (2012). Novel antimicrobial polyethylene composites prepared by metallocenic in situ polymerization with TiO_2_--based nanoparticles. J. Polym. Sci. Part A Polym. Chem..

[52] Bartelmess J., Giordani S. (2014). Carbon nano-onions (multi-layer fullerenes): Chemistry and applications. Beilstein J. Nanotechnol..

[53] Gholampour A., Valizadeh Kiamahalleh M., Tran D.N.H., Ozbakkaloglu T., Losic D. (2017). From Graphene Oxide to Reduced Graphene Oxide: Impact on the Physiochemical and Mechanical Properties of Graphene-Cement Composites. ACS Appl. Mater. Interfaces.

[54] Sánchez-Valdes S., Zapata-Domínguez A.G., Martinez-Colunga J.G., Mendez-Nonell J., Ramos de Valle L.F., Espinoza-Martinez A.B., Morales-Cepeda A., Lozano-Ramirez T., Lafleur P.G., Ramirez-Vargas E. (2018). Influence of functionalized polypropylene on polypropylene/graphene oxide nanocomposite properties. Polym. Compos..

[55] Ruiz S., Tamayo A.J., Delgado Ospina J., Navia Porras P.D., Valencia Zapata E.M., Mina Hernandez H.J., Valencia H.C., Zuluaga F., Grande Tovar D.C. (2019). Antimicrobial Films Based on Nanocomposites of Chitosan/Poly(vinyl alcohol)/Graphene Oxide for Biomedical Applications. Biomolecules.

[56] Kulkarni V.H., Kulkarni P.V., Keshavayya J. (2007). Glutaraldehyde--crosslinked chitosan beads for controlled release of diclofenac sodium. J. Appl. Polym. Sci..

[57] Ngah W.S.W., Fatinathan S. (2008). Adsorption of Cu (II) ions in aqueous solution using chitosan beads, chitosan–GLA beads and chitosan–alginate beads. Chem. Eng. J..

[58] Monteiro O.A.C., Airoldi C. (1999). Some studies of crosslinking chitosan–glutaraldehyde interaction in a homogeneous system. Int. J. Biol. Macromol..

[59] Mallakpour S., Zadehnazari A. (2014). A facile, efficient, and rapid covalent functionalization of multi-walled carbon nanotubes with natural amino acids under microwave irradiation. Prog. Org. Coatings.

[60] Feng F., Liu Y., Zhao B., Hu K. (2012). Characterization of half N-acetylated chitosan powders and films. Procedia Eng..

[61] Samuels R.J. (1981). Solid state characterization of the structure of chitosan films. J. Polym. Sci. Polym. Phys. Ed..

[62] Salmon S., Hudson S.M. (1997). Crystal morphology, biosynthesis, and physical assembly of cellulose, chitin, and chitosan. J. Macromol. Sci. Part C Polym. Rev..

[63] Goumri M., Poilâne C., Ruterana P., Doudou B.B., Wéry J., Bakour A., Baitoul M. (2017). Synthesis and characterization of nanocomposites films with graphene oxide and reduced graphene oxide nanosheets. Chinese J. Phys..

[64] Fu G., Vary P.S., Lin C.-T. (2005). Anatase TiO_2_ nanocomposites for antimicrobial coatings. J. Phys. Chem. B.

[65] Amin S.A., Pazouki M., Hosseinnia A. (2009). Synthesis of TiO_2_–Ag nanocomposite with sol-gel method and investigation of its antibacterial activity against *E. coli*. Powder Technol..

[66] Khanna P.K., Singh N., Charan S. (2007). Synthesis of nanoparticles of anatase-TiO_2_ and preparation of its optically transparent film in PVA. Mater. Lett..

[67] Mahshid S., Askari M., Ghamsari M.S. (2007). Synthesis of TiO_2_ nanoparticles by hydrolysis and peptization of titanium isopropoxide solution. J. Mater. Process. Technol..

[68] Sangeetha K., Angelin Vinodhini P., Sudha P.N., Alsharani Faleh A., Sukumaran A. (2019). Novel chitosan based thin sheet nanofiltration membrane for rejection of heavy metal chromium. Int. J. Biol. Macromol..

[69] Tang C., Chen N., Zhang Q., Wang K., Fu Q., Zhang X. (2009). Preparation and properties of chitosan nanocomposites with nanofillers of different dimensions. Polym. Degrad. Stab..

[70] Pandele A.M., Ionita M., Crica L., Dinescu S., Costache M., Iovu H. (2014). Synthesis, characterization, and in vitro studies of graphene oxide/chitosan-polyvinyl alcohol films. Carbohydr. Polym..

[71] Yang X., Tu Y., Li L., Shang S., Tao X. (2010). Well-Dispersed Chitosan/Graphene Oxide Nanocomposites. ACS Appl. Mater. Interfaces.

[72] Celebi H., Kurt A. (2015). Effects of processing on the properties of chitosan/cellulose nanocrystal films. Carbohydr. Polym..

[73] Monier M., Ayad D.M., Wei Y., Sarhan A.A. (2010). Immobilization of horseradish peroxidase on modified chitosan beads. Int. J. Biol. Macromol..

[74] Ma Q., Liang T., Cao L., Wang L. (2018). Intelligent poly (vinyl alcohol)-chitosan nanoparticles-mulberry extracts films capable of monitoring pH variations. Int. J. Biol. Macromol..

[75] Yadav I., Nayak S.K., Rathnam V.S.S., Banerjee I., Ray S.S., Anis A., Pal K. (2018). Reinforcing effect of graphene oxide reinforcement on the properties of poly (vinyl alcohol) and carboxymethyl tamarind gum based phase-separated film. J. Mech. Behav. Biomed. Mater..

[76] Li S., Pan X., Wallis L.K., Fan Z., Chen Z., Diamond S.A. (2014). Comparison of TiO_2_ nanoparticle and graphene–TiO_2_ nanoparticle composite phototoxicity to Daphnia magna and Oryzias latipes. Chemosphere.

[77] Tomihata K., Ikada Y. (1997). In vitro and in vivo degradation of films of chitin and its deacetylated derivatives. Biomaterials.

[78] Pella M.C.G., Lima-Tenório M.K., Tenorio-Neto E.T., Guilherme M.R., Muniz E.C., Rubira A.F. (2018). Chitosan-based hydrogels: From preparation to biomedical applications. Carbohydr. Polym..

[79] Fujita M., Ishihara M., Simizu M., Obara K., Ishizuka T., Saito Y., Yura H., Morimoto Y., Takase B., Matsui T. (2004). Vascularization in vivo caused by the controlled release of fibroblast growth factor-2 from an injectable chitosan/non-anticoagulant heparin hydrogel. Biomaterials.

[80] Pawar V., Bulbake U., Khan W., Srivastava R. (2019). Chitosan sponges as a sustained release carrier system for the prophylaxis of orthopedic implant-associated infections. Int. J. Biol. Macromol..

[81] Rinaudo M., Milas M., Le Dung P. (1993). Characterization of chitosan. Influence of ionic strength and degree of acetylation on chain expansion. Int. J. Biol. Macromol..

[82] Mangadlao J.D., De Leon A.C.C., Felipe M.J.L., Cao P., Advincula P.A., Advincula R.C. (2015). Grafted carbazole-assisted electrodeposition of graphene oxide. ACS Appl. Mater. Interfaces.

[83] Fan J., Grande C.D., Rodrigues D.F. (2017). Biodegradation of graphene oxide-polymer nanocomposite films in wastewater. Environ. Sci. Nano.

[84] Grande C.D., Mangadlao J., Fan J., De Leon A., Delgado-Ospina J., Rojas J.G., Rodrigues D.F., Advincula R. (2017). Chitosan Cross-Linked Graphene Oxide Nanocomposite Films with Antimicrobial Activity for Application in Food Industry. Macromol. Symp..

[85] Valencia Zapata E.M., Mina Hernandez H.J., Grande Tovar D.C., Valencia Llano H.C., Diaz Escobar A.J., Vázquez-Lasa B., San Román J., Rojo L. (2019). Novel Bioactive and Antibacterial Acrylic Bone Cement Nanocomposites Modified with Graphene Oxide and Chitosan. Int. J. Mol. Sci..

[86] Zhao F., Yu B., Yue Z., Wang T., Wen X., Liu Z., Zhao C. (2007). Preparation of porous chitosan gel beads for copper ( II ) ion adsorption. J. Hazard. Mater..

[87] Jeon C., Wolfgang H.H. (2003). Chemical modification of chitosan and equilibrium study for mercury ion removal. Water Res..

[88] Nara S., Komiya T. (1983). Studies on the Relationship Between Water-satured State and Crystallinity by the Diffraction Method for Moistened Potato Starch. Starke.

