# Supplementary material for: Synthesis of Chitosan Beads Incorporating Graphene Oxide/Titanium Dioxide Nanoparticles for In Vivo Studies
Source: Molecules. 2020 May 14;25(10):2308. doi: 10.3390/molecules25102308 (PMC7287625; doi:10.3390/molecules25102308)
Supplement: Supplementary file 1 [file molecules-25-02308-s001.pdf]

Supporting information of

Synthesis of Chitosan Beads Incorporating Graphene Oxide/Titanium Dioxide Nanoparticles for In Vivo Studies

Carlos David Grande Tovar[1]*, Jorge Iván Castro[2], Carlos Humberto Valencia[3], Paula A. Zapata[4], Edwin Florez López[5], Manuel N. Chaur[2,6]* Mayra Eliana Valencia Zapata[7] and José Herminsul Mina Hernandez[7]

1. Programa de Química, Universidad del Atlántico, Carrera 30 Número 8-49, Puerto Colombia 081008, Colombia; carlosgrande@mail.uniatlantico.edu.co (C.D.G.T)
2. Grupo de Investigación SIMERQO, Departamento de Química, Universidad del Valle, Calle 13 No. 100-00, 76001 Cali, Colombia; jorgecastro@correounivalle.edu.co; manuel.chaur@correounivalle.edu.co (M.N.C.) (J.I.C.)
3. Escuela de Odontología, Grupo biomateriales dentales, Universidad del Valle, Calle 13 No. 100-00, 76001 Cali, Colombia; carlos.humberto.valencia@correounivalle.edu.co (C.H.V.)
4. Grupo de Polímeros, Facultad de Química y Biología, Universidad de Santiago de Chile, USACH, Casilla 40, Correo 33, 9170020, Santiago, Chile paula.zapata@usach.cl (P.A.Z.)
5. Grupo de Investigación en Química y Biotecnología QUIBIO, Universidad Santiago de Cali, Calle 5 No 62-00, 760035 Cali, Colombia; edwin.florez00@usc.edu.co (E.F.L.)
6. Centro de Excelencia en Nuevos Materiales (CENM), Universidad del Valle, Calle 13 No. 100-00, Santiago de Cali 760032, Colombia; manuel.chaur@correounivalle.edu.co (M.N.C.)
7. Escuela de Ingeniería de Materiales, Facultad de Ingeniería, Universidad del Valle, Calle 13 No. 100-00, Santiago de Cali 760032, Colombia; valencia.mayra@correounivalle.edu.co (M.E.V.); jose.mina@correounivalle.edu.co (J.H.M.)

- Correspondence: carlosgrande@mail.uniatlantico.edu.co (C.D.G.T); manuel.chaur@correounivalle.edu.co (M.N.C.)

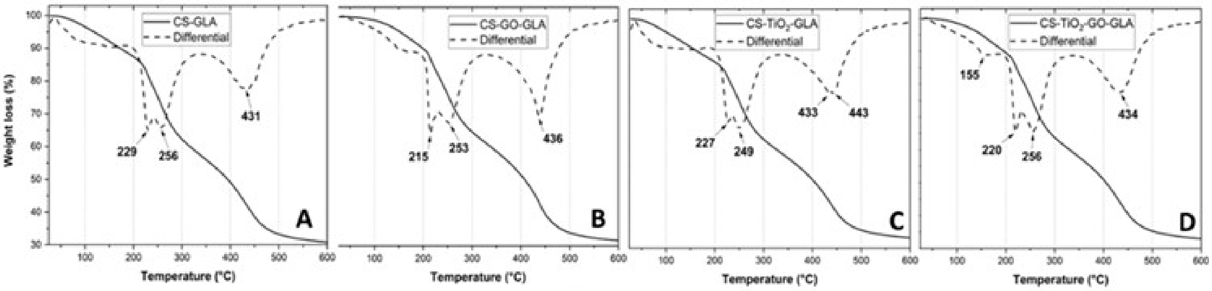

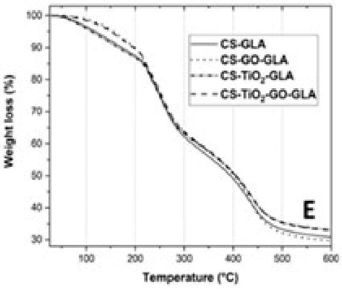

Figure S1. TGA and DTG curves of the nanocomposite beads A) CS-GLA, B) CS-GO-GLA, C) CS-TiO_2-GLA, D) CS-TiO_2-GO-GLA, E) TGA of all the samples

Table S1. Percentage resorption of nanocomposite CS beads after three months of implantation

**Sample**	**Resorption area (µm)**	**Total area (µm)**	**% resorption**
CS-GLA	0.058±0.001	32.5	0.18
CS-GO-GLA	0.002±0.002	34.3	0.006
CS-TiO_2-GLA	0.08±0.002	32.6	0.25
CS-GO-TiO_2-GLA	1.41±0.012	33.4	4.2
